# LncRNA THRIL is upregulated in sepsis and sponges miR-19a to upregulate TNF-α in human bronchial epithelial cells

**DOI:** 10.1186/s12950-020-00259-z

**Published:** 2020-09-10

**Authors:** Tao Liu, Jingbin Liu, Chunhua Tian, Hongyuan Wang, Min Wen, Mingyu Yan

**Affiliations:** 1 Department of Respiratory and Critical Care Medicine, Inner Mongolia Baogang Hospital (The Third Affiliated Hospital of Inner Mongolia Medical University), Baotou, 014032 Inner Mongolia China; 2 Department of Respiratory Medicine, Hospital of FIRMACO (The Fourth Affiliated Hospital of Inner Mongolia Medical University), Baotou, 014032 Inner Mongolia China; 3 Department of Nephrology, Inner Mongolia Baogang Hospital (The Third Affiliated Hospital of Inner Mongolia Medical University), No.20 Shaoxian Road, Kundulun District, Baotou City, 014032 Inner Mongolia China; 4 Department of Dental department, Hospital of FIRMACO (The Fourth Affiliated Hospital of Inner Mongolia Medical University), Baotou, 014032 Inner Mongolia China; 5 Department of Pharmacy, Hospital of FIRMACO (The Fourth Affiliated Hospital of Inner Mongolia Medical University), Baotou, 014032 Inner Mongolia China

**Keywords:** Sepsis, lncRNA THRIL, miR-19a, TNF-α, Apoptosis

## Abstract

**Background:**

Long non-coding RNAs (lncRNAs) have been demonstrated to play critical roles in various diseases. Our bioinformatics analysis showed that lncRNA TNFα and heterogenous nuclear ribonucleoprotein L (hnRNPL) related immunoregulatory LincRNA (THRIL) may interact with miR-19a, which targets TNF-α. This study aimed to explore the role of THRIL, an enhancer of LPS-induced inflammatory, in sepsis.

**Methods:**

Research subjects of the present study included 66 sepsis patients and 66 healthy volunteers. The expression levels of THRIL, miR-19a and TNF-α in plasma samples from these participants were determined by RT-qPCR. The interaction between THRIL and miR-19a was explored by performing overexpression experiments in human bronchial epithelial cells (HBEpCs). The roles of THRIL, miR-19a and TNF-α in regulating the apoptosis of HBEpCs were analyzed by cell apoptosis assay.

**Results:**

We found that THRIL was upregulated in sepsis patients. THRIL is predicted to interact with miR-19a, and the interaction was confirmed by dual-luciferase activity assay. However, THRIL and miR-19a did not affect the expression of each other. Instead, overexpression of THRIL resulted in the increased expression levels of TNF-α, a downstream target of miR-19a in HBEpCs. In HBEpCs, LPS treatment induced the overexpression of THRIL. Cell apoptosis analysis showed that overexpression of THRIL and TNF-α promoted the apoptosis of HBEpCs induced by LPS, while overexpression of miR-19a played an opposite role. Overexpression of THRIL attenuated the effects of overexpression of miR-19a.

**Conclusion:**

Therefore, THRIL is upregulated in sepsis and may sponge miR-19a to upregulate TNF-α, thereby promoting lung cell apoptosis.

## Background

Sepsis is the body’s extreme reactions to infection that can result in organ failure, tissue damage or even death if treatment was not performed properly and timely [1]. In severe cases, mortality rate caused by sepsis may reach 50% [2, 3]. Sepsis affects most important organs, such as kidney, heart and lung, mainly by inducing cell injuries and apoptosis [4, 5]. Therefore, prevention of cell apoptosis is considered as a promising target for anti-sepsis therapy [4, 5]. However, molecular mechanism of the cell apoptosis induced by sepsis remains unclear [6], resulting in difficulties in the development of novel therapeutic approaches.

Studies on the molecular pathogenesis of sepsis have revealed a large number of molecular pathways involved in this disease [7]. Understanding the functionality of these molecular players in sepsis provided novel insights into the development of targeted therapies [8]. Extensive studies have revealed that non-coding RNAs (ncRNAs), such as long (> 200 nt) ncRNAs (lncRNAs) and microRNAs (miRNAs), do not encode protein products but play critical roles in diverse biological processes by regulating protein synthesis [9]. In effect, regulating the expression of critical ncRNA players in sepsis may benefit the treatment of this disease [10]. However, the functions of most ncRNAs remain unclear. LncRNA THRIL (TNFα and heterogenous nuclear ribonucleoprotein L (hnRNPL) related immunoregulatory LincRNA) is an enhancer of LPS-induced inflammatory [11]. In another study, THRIL was demonstrated to increase the risk of acute respiratory distress syndrome (ARDS) and was positively correlated with inflammatory responses, disease severity and mortality in sepsis patients [12]. Our bioinformatics analysis showed that THRIL may interact with miR-19a, which targets TNF-α [13], a critical player in sepsis [14]. This study was therefore performed to investigate the interactions among THRIL, miR-19a and TNF-α in sepsis.

## Methods

### Sepsis patients and healthy controls

Research subjects of the present study included 66 sepsis patients (40 males and 26 females, 42 to 66 years old, mean age 54.2 ± 6.7 years old) and 66 healthy volunteers (40 males and 26 females, 42 to 66 years old, mean age 54.0 ± 7.0 years old). All participants were enrolled at the Inner Mongolia Baogang Hospital between December 2017 and December 2018. This study was approved by the Ethics Committee of this hospital. No therapy was initiated before the admission of patients. All patients were diagnosed with sepsis for the first time. No previous history of severe diseases, such as cancers, diabetes and heart diseases were observed. All healthy controls were selected at the physiological healthy center of aforementioned hospital after they received systemic physiological examinations. All physiological functions of the healthy controls were normal. All participants signed the written informed consent.

### Plasma preparations

After fasting for overnight, blood extraction (5 ml) was performed on all patients (before the use of antibiotics) and controls. Blood samples were mixed with EDTA and were centrifuged (1200x g) at room temperature for 10 min to separate plasma. Plasma samples were stored in liquid nitrogen.

### Human bronchial epithelial cells (HBEpCs)

All cell experiments were performed using HBEpCs (Sigma-Aldrich). Cell culture was performed following the manufacturers’ instructions. All passage 4–6 generations, cells were collected at about 85% confluence to be used in following experiments.

### RNA-RNA interaction prediction

The potential base paring formed by THRIL and miR-19a was predicted by IntaRNA 2.0 (http://rna.informatik.uni-freiburg.de/IntaRNA/Input.jsp). THRIL was used as long sequence and miR-19a was used as short sequence. All other parameters were default.

### Transient transfections and dual luciferase reporter assay

Expression vector of THRIL or TNF-α was constructed using pcDNA 3.1 vector (Invitrogen) as the backbone. Negative control (NC) miRNA, miR-19a mimic, miR-19a inhibitor, and THRIL siRNA (siTHRIL) were purchased from Sigma-Aldrich (USA). HBEpCs were transfected with 10 nM expression vector (10 nM) or miRNA mimic (40 nM) and their relevant siRNA or inhibitor using Lipofectamine 2000 (Invitrogen). Untransfected cells were used as the control (C) cells. NC cells were empty vector- or NC miRNA- transfected cells. To perform dual-luciferase reporter assay, THRIL luciferase reporter vector was constructed using pmirGLO vector (Promega, USA). HBEpCs were co-transfected with either THRIL vector and miR-19a (miR-19a group) or THRIL vector and NC miRNA (NC group) using lipofectamine 2000. Dual-Luciferase Reporter Assay System (Promega) was used to measure luciferase activity 48 h post-transfection.

### RNA preparations

Total RNAs were isolated from HBEpCs and plasma samples using Trizol reagent (Invitrogen), following by digestion with DNase I to remove genomic RNAs. RNA precipitation and washing steps were performed using 85% ethanol. The RNA concentrations were measured using the Thermo Fisher NanoDrop 2000 Spectrophotometer. To assess the effects of LPS (L2630-25MG, Sigma-Aldrich) treatment on the expression of THRIL, RNA isolation from HBEpCs was performed after treatment with 0, 2, 5 or 10 μg/ml LPS for 24 h.

### RT-qPCR assay

BlazeTaq™ One-Step SYBR Green RT-qPCR Kit was used to prepare PCR reactions. The expression levels of THRIL and TNF-α were measured with GAPDH as endogenous control. The measurement of the expression of mature miR-19a was performed using All-in-One™ miRNA qRT-PCR reagent kit (GeneCopoeia). All operations were performed following the manufacturers’ instructions. PCR reactions were repeated 3 times and the 2^-ΔΔCT^ method was used to perform data analysis.

### Western blotting

The isolation of total proteins from HBEpCs was performed using RIPA solution (Sigma-Aldrich), followed by BCA assay to measure protein concentrations. All protein samples were incubated at 95 °C for 10 min to denature proteins, followed by separating protein samples (35 μg protein per lane) using 10% SDS-PAGE gel. PVDF membranes were used to transfer proteins and blocking in 5% non-fat milk (FBS) at room temperature for 2 h. After that, primary antibodies including rabbit anti-TNF-α (ab9635, Abcam) and GAPDH (ab9483, Abcam) were used to incubate with membranes at 4 °C for 12 h. After that, HRP Goat Anti-Rabbit (IgG) (ab6721, Abcam) secondary antibody was used to incubate with the membranes at room temperature for 2 h. Data normalization was performed using ImageJ v1.48.

### Cell apoptosis assay

HBEpCs were harvested at 48 h, followed by incubation in medium containing 10 μg/ml LPS for another 48 h. After that, cells were harvested and washed with pre-cold PBS. PI and FITC-annexin V staining were performed and flow cytometry was used to separate apoptotic cells.

### Statistical analysis

Data from 3 independent biological replicates were expressed as mean ± SEM. Comparisons between two groups were performed using unpaired t test. Comparisons among multiple groups were performed using ANOVA (one-way) and Tukey test. *P* < 0.05 was considered to be statistically significant.

## Results

### Expression of THRIL, miR-19a and TNF-α were altered in sepsis patients

**Fig. 1:**
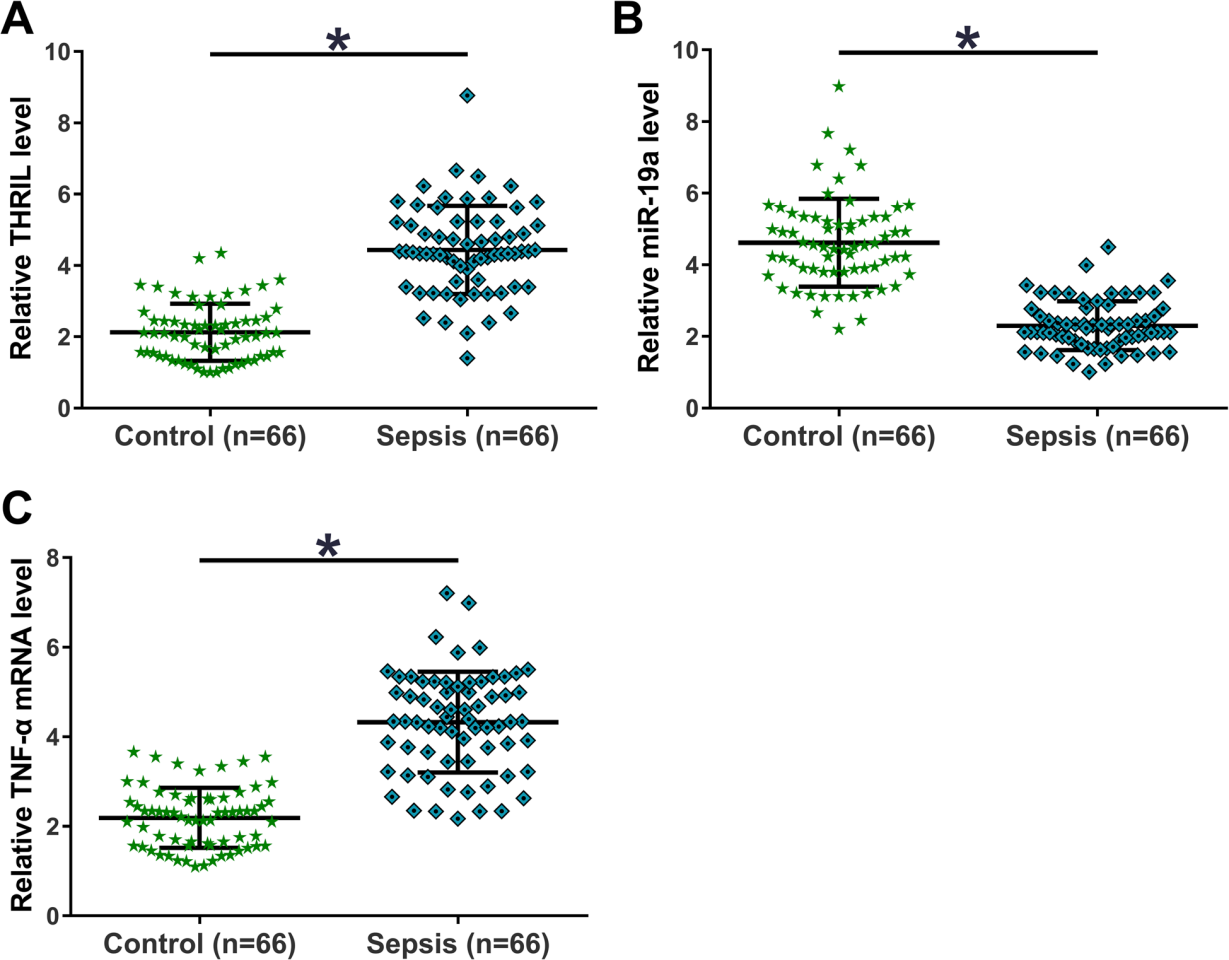
Expression of of THRIL, miR-19a and TNF-α mRNA were altered in sepsis patients. Expression levels of THRIL (**a**), miR-19a (**b**) and TNF-α mRNA (**c**) in plasma from sepsis patients (*n* = 66) and healthy controls (*n* = 66) were measured by RT-qPCR assay. PCR reactions were repeated 3 times and mean ± Standard Deviation (SD) values were presented.*, *p* < 0.05

The expression levels of THRIL, miR-19a and TNF-α in plasma from sepsis patients (*n* = 66) and healthy controls (*n* = 66) were measured using RT-qPCR assay. Compared to the control group, significantly upregulated THRIL was observed in sepsis patients (Fig. 1a, *p* < 0.05). Moreover, compared to the control groups, sepsis group exhibited significantly lower expression levels of miR-19a (Fig. 1b, *p* < 0.05) and significantly higher expression levels of TNF-α (Fig. 1c, *p* < 0.05). These data suggested that THRIL, miR-19a and TNF-α may participate in sepsis.

### THRIL and miR-19a interacted with each other but did not regulate the expression of each other

**Fig. 2:**
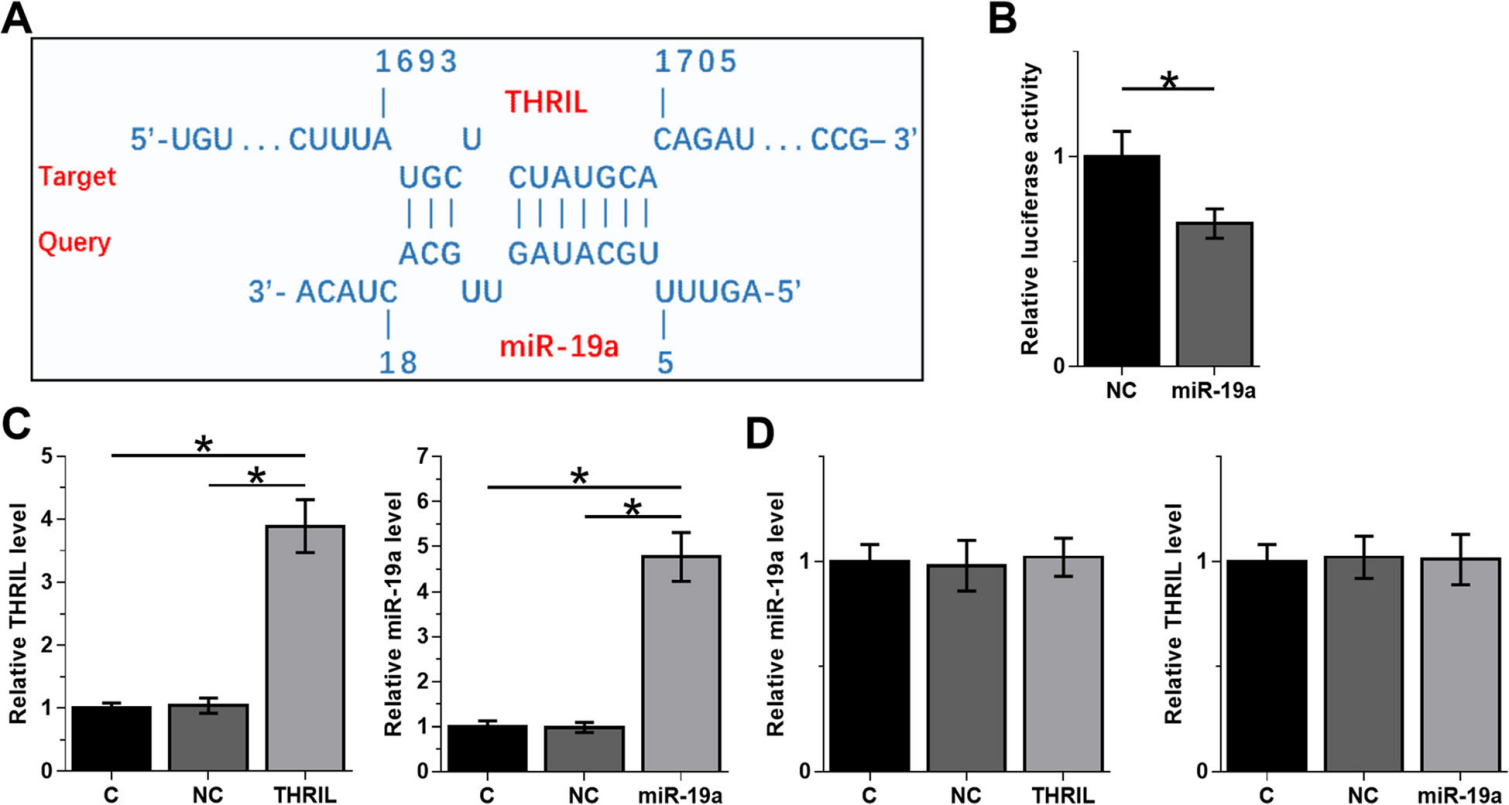
THRIL and miR-19a interacted with each other but did not regulate the expression of each other. The potential interaction between THRIL and miR-19a was predicted by IntaRNA 2.0. It was observed that THRIL and miR-19a may form strong base pairing (**a**). Dual luciferase reporter assay was used analyze the interaction between THRIL and miR-19a. HBEpCs were co-transfected with either THRIL vector and miR-19a (miR-19a group) or THRIL vector and NC miRNA (NC group). Luciferase activity was measured at 48 h post-transfection and was compared between two groups (**b**). HBEpCs were transfected with THRIL expression vector or miR-19a mimic to explore the interaction between them. The overexpression of THRIL and miR-19a was confirmed at 48 h post-transfection (**c**). The effects of the overexpression of THRIL and miR-19a on the expression of each other were also analyzed by RT-qPCR at 48 h post-transfection (**d**). All experiments were repeated 3 times and mean ± SD values were presented. *, *p* < 0.05

The potential interaction between THRIL and miR-19a was predicted by IntaRNA 2.0. It was observed that THRIL and miR-19a may form strong base pairing (Fig. 2a). Dual luciferase reporter assay was used to evaluate the interaction between THRIL and miR-19a. Compared to NC group (see materials and methods section for grouping details), significantly lower relative luciferase activity was observed in miR-19a group (Fig. 2b, *p* < 0.05). Therefore, THRIL and miR-19a can directly interact with each other. HBEpCs were transfected with THRIL expression vector or miR-19a mimic to perform further analysis. The overexpression of THRIL and miR-19a was confirmed at 48 h post-transfection (Fig. 2c, *p* < 0.05). Compared to NC and C groups, overexpression of THRIL and miR-19a did not significantly affect the expression of each other (Fig. 2d). As previous studies showed that THRIL interacts with hnRNPL to regulate TNFa induction, so we further investigated whether THRIL and miR-19a would affect the expression levels of hnRNPL in HBEpCs. However, we found that overexpression of THRIL and miR-19a could not change the expression of hnRNPL (suppl. Fig. 1A-B), which implicated that THRIL or miR-19a have no correlation with the expression of hnRNPL.

### Overexpression of THRIL resulted in the increased expression levels of TNF-α

**Fig. 3:**
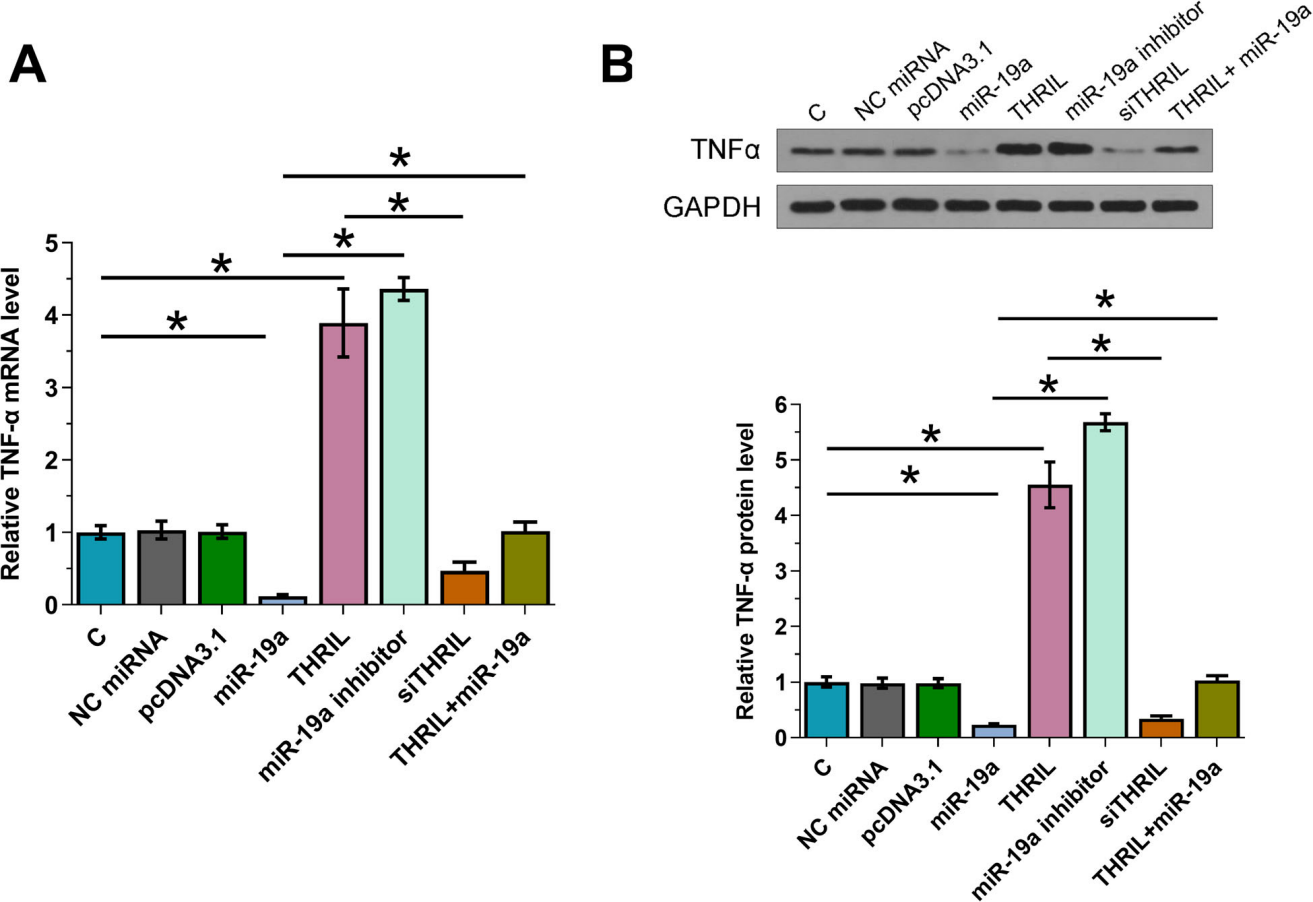
Overexpression of THRIL resulted in increased expression levels of TNF-α. Data presented above indicated the potential role of THRIL as the endogenous sponge of miR-19a. To test this possibility, the effects of THRIL and miR-19a on TNF-α, a downstream target of miR-19a, were analyzed by both RT-qPCR (**a**) and western blot (**b**). All experiments were repeated 3 times and mean ± SD values were presented. *, *p* < 0.05. (Note: C: untransfected control cells; siTHRIL: THRIL siRNA)

Data presented above indicated the potential role of THRIL as an endogenous sponge of miR-19a. To test this possibility, the effects of overexpression of THRIL and miR-19a on the expression of TNF-α, a downstream target of miR-19a, were analyzed by both RT-qPCR (Fig. 3a) and western blot (Fig. 3b). It was observed that overexpression of miR-19a resulted in downregulated TNF-α, further confirming the targeting of TNF-α by miR-19a. In contrast, overexpression of THRIL resulted in upregulated TNF-α and reduced the inhibitory effects of miR-19a on TNF-α (*p* < 0.05).

### THRIL regulated the miR-19a/TNF-α axis to promote the apoptosis of HBEpCs

**Fig. 4:**
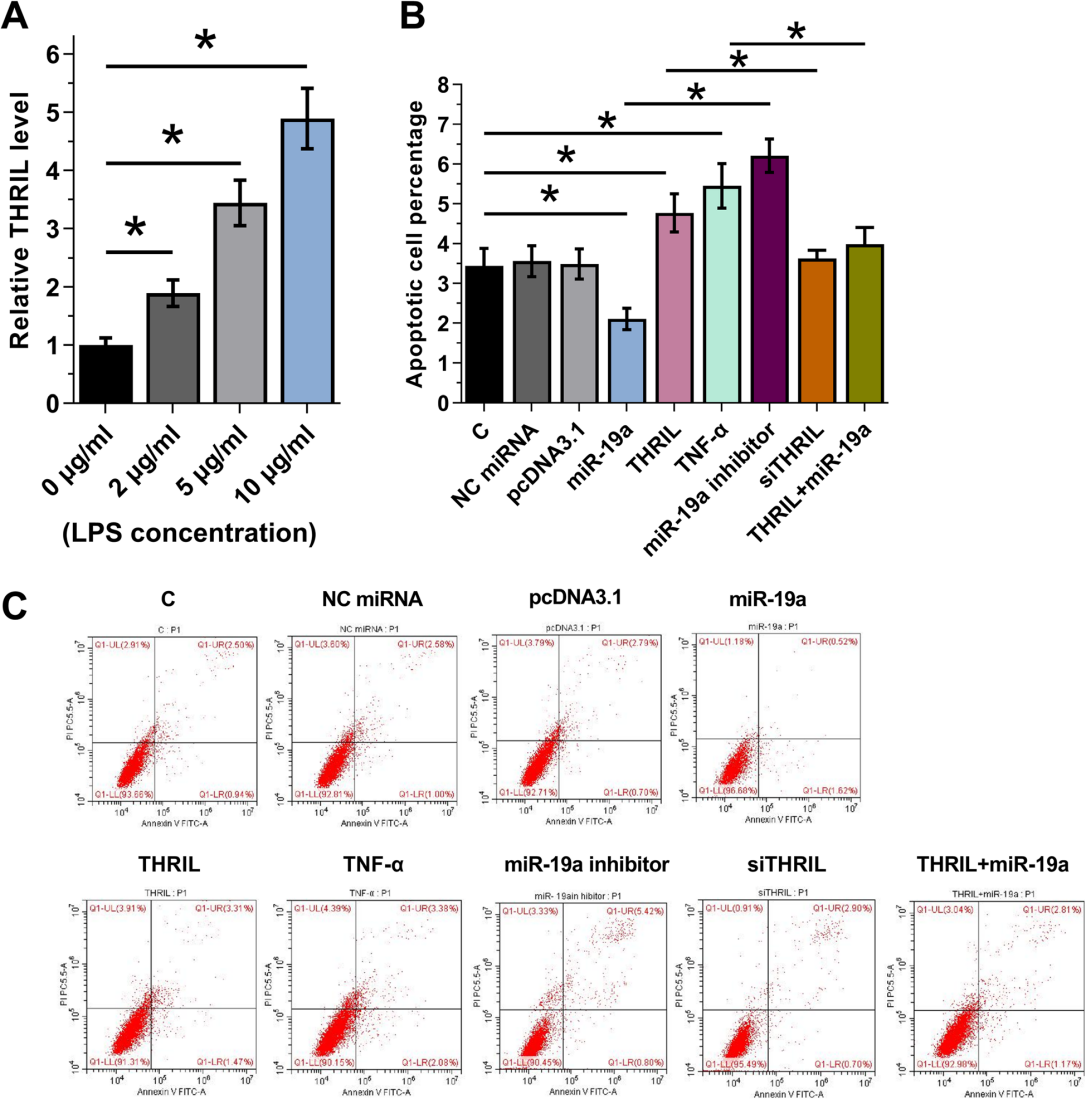
THRIL regulated miR-19a/TNF-α axis to promote the apoptosis of HBEpCs. HBEpCs cultivated in medium supplemented with 0, 2, 5 or 10 μg/ml LPS for 24 h, followed by the measurement of the expression levels of THRIL by performing RT-qPCR (**a**). The effects of THRIL, miR-19a and TNF-αon the apoptosis of HBEpCs were analyzed by cell apoptosis assay after the treatment with 10 μg/ml LPS for 48 h (**b**). All experiments were repeated 3 times and mean ± SD values were presented. *, *p* < 0.05. (Note: C: untransfected control cells; siTHRIL: THRIL siRNA)

HBEpCs were cultivated in medium supplemented with 0, 2, 5 or 10 μg/ml LPS for 24 h, followed by the measurement of expression levels of THRIL by performing RT-qPCR. Compared to C and NC groups, LPS treatment upregulated the expression of THRIL in a dose-dependent manner (Fig. 4a, *p* < 0.05). Interestingly, we also detected the cell viability after treatment with 0–20 μg/ml LPS for 24 h using trypan blue living cell count, cell viability had no significantly change below 10 μg/ml (suppl. Fig. 2) The effects of overexpression of THRIL, miR-19a and TNF-α on the apoptosis of HBEpCs were assessed by cell apoptosis assay. Compared to C and NC groups, overexpression of THRIL and TNF-α promoted the apoptosis of HBEpCs induced by LPS, while overexpression of miR-19a inhibited cell apoptosis. Moreover, overexpression of THRIL attenuated the effects of overexpression of miR-19a. However, the effects of THRIL and miR-19a would also be reversed by its relevant siRNA and inhibitor (Fig. 4b-c, *p* < 0.05).

## Discussion

This study mainly investigated the interactions among THRIL, miR-19a and TNF-α in sepsis. We found that THRIL was upregulated in sepsis and may upregulate TNF-α by sponging miR-19a to promote cell apoptosis induced by LPS.

THRIL has been proven to participate in multiple pathological processes [11, 15]. For instance, silencing of THRIL leads to altered expression of miR-99a, which in turn suppresses hypoxia-induced injuries in H9C2 cells [15]. In a recent study, THRIL was demonstrated to be an enhancer of LPS-induced inflammatory injury in ATDC5 cells mainly by reducing the expression levels of miR-125b [11]. It is well-known that LPS-induced inflammatory can promote the progression of sepsis [16]. Therefore, THRIL may also participate in sepsis. To the best of our knowledge, this study is the first to report the upregulation of THRIL in sepsis. In addition, we also revealed that LPS treatment could upregulate the expression of THRIL in HBEpCs, which in turn promoted the apoptosis of lung cells. Therefore, LPS-induced THRIL may participate in sepsis by inducing cell apoptosis.

It has been reported that miR-19a and CD22 could form a feedback regulation loop to regulate the responses of B cells in sepsis [17]. MiR-19a may also participate in sepsis by targeting TNF-α [13], which a critical player in sepsis [14]. Our study confirmed the targeting of TNF-α by miR-19a. In addition, our study is the first to show that miR-19a can target TNF-α to suppress LPS-induced lung cell apoptosis.

The key finding of this study is that THRIL may serve as an endogenous sponge of miR-19a to attenuate its inhibitory effects on cell apoptosis-induced by LPS and the expression of TNF-α. Therefore, we characterized a novel THRIL/miR-19a/TNF-α pathway in sepsis. However, it also has been reported that THRIL may also regulate the expression of TNFα by interacting with hnRNPL [18]. Therefore, THRIL may regulate the expression of TNFα through multiple ways.

This study is limited by the small sample size. In addition, in vivo animal model experiments were not performed in this study. Our future studies will include more patients and perform sepsis animal model experiments to further confirm our conclusions. Sepsis induces a series of disorders in major organs. The expression of THRIL may also be affected by those complications. Our future studies will explore the effects of sepsis-related complications on the expression of THRIL.

## Conclusion

In conclusion, THRIL is upregulated in sepsis and may sponge miR-19a to upregulate TNF-α, thereby promoting cell apoptosis induced by LPS.

## Supplementary information

**Additional file 1: Suppl Figure 1.** THRIL and miR-19a did not regulate the expression of hnRNPL in HBEpCs. RT-qPCR detect hnRNPL expression after 48 h transfection of THRIL and miR-19a into HBEpCs, respectively. (A) Overexpression of THRIL could not change the expression of hnRNPL; (B) MiR-19a could not regulate the expression of hnRNPL. (Note: C: untransfected control cells).

**Additional file 2: Suppl Figure 2**. Lower concentration of LPS would not affect HBEpCs cell viability. The cell viability after treatment with 0–20 μg/ml LPS for 24 h was analyzed by trypan blue living cell count, cell viability had no significantly change below 10 μg/ml (*, *p* < 0.05).

## Data Availability

The datasets used and/or analyzed during the current study are available from the corresponding author on reasonable request.

## Abbreviations

HBEpCs: human bronchial epithelial cells
lncRNA TNFα and heterogenous nuclear ribonucleoprotein L (hnRNPL) related THRIL: immunoregulatory LincRNA
ncRNAs: non-coding RNAs
lncRNAs: long ncRNAs
