# Supplementary figures and images for: LncRNA THRIL is upregulated in sepsis and sponges miR-19a to upregulate TNF-α in human bronchial epithelial cells
Source: J Inflamm (Lond). 2020 Sep 10;17:31. doi: 10.1186/s12950-020-00259-z (PMC7488348; doi:10.1186/s12950-020-00259-z)

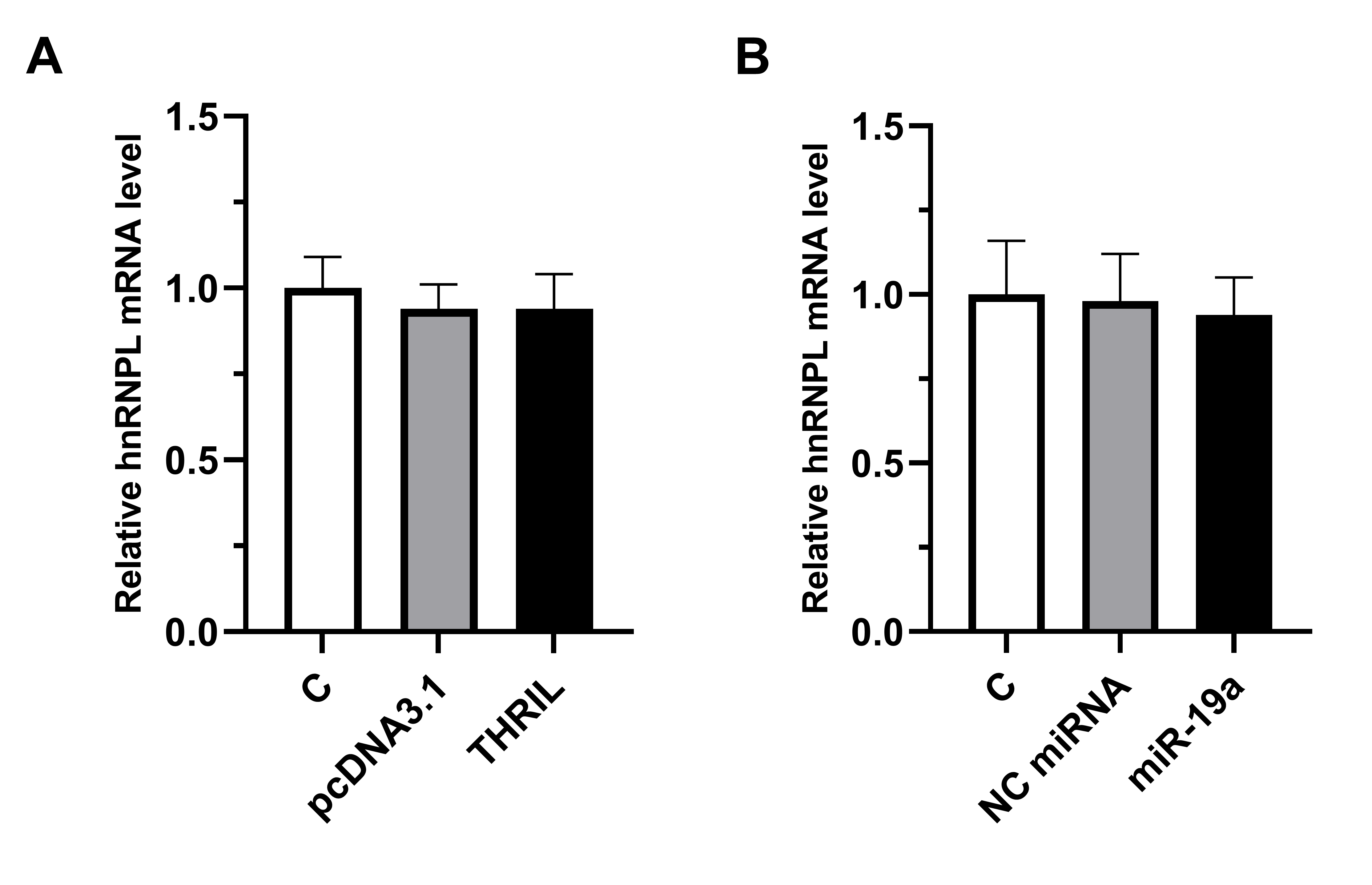

Supplement: Supplementary file 1 — Additional file 1: Suppl Figure 1. THRIL and miR-19a did not regulate the expression of hnRNPL in HBEpCs. RT-qPCR detect hnRNPL expression after 48 h transfection of THRIL and miR-19a into HBEpCs, respectively. (A) Overexpression of THRIL could not change the expression of hnRNPL; (B) MiR-19a could not regulate the expression of hnRNPL. (Note: C: untransfected control cells). [file 12950_2020_259_MOESM1_ESM.tif]

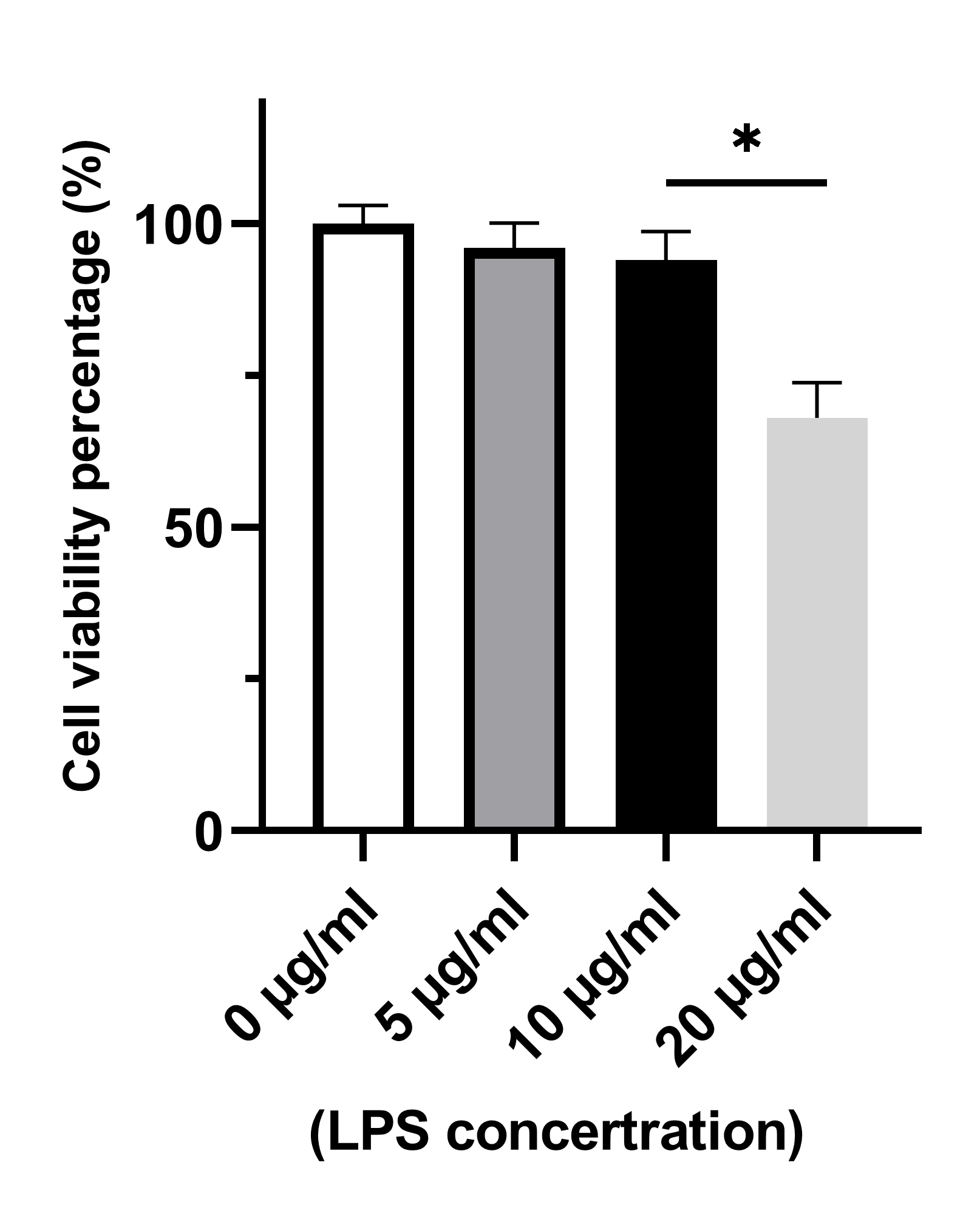

Supplement: Supplementary file 2 — Additional file 2: Suppl Figure 2. Lower concentration of LPS would not affect HBEpCs cell viability. The cell viability after treatment with 0–20 μg/ml LPS for 24 h was analyzed by trypan blue living cell count, cell viability had no significantly change below 10 μg/ml (*, p < 0.05). [file 12950_2020_259_MOESM2_ESM.tif]
